# Perceptions of cardiac rehabilitation patients, specialists and rehabilitation programs regarding cardiac rehabilitation wait times

**DOI:** 10.1186/1472-6963-12-259

**Published:** 2012-08-16

**Authors:** Sherry L Grace, Yongyao Tan, Louise Marcus, William Dafoe, Chris Simpson, Neville Suskin, Caroline Chessex

**Affiliations:** 1 York University & University Health Network, Toronto, Ontario, Canada; 2 University Health Network, Toronto, Ontario, Canada; 3 Canadian Cardiovascular Society, Ottawa, Ontario, Canada; 4 University of Alberta & Alberta Health Services, Edmonton, Alberta, Canada; 5 Kingston General Hospital, Kingston, Ontario, Canada; 6 London Health Sciences Centre & University of Western Ontario, London, Ontario, Canada

## Abstract

**Background:**

In 2006, the Canadian Cardiovascular Society (CCS) Access to Care Working Group recommended a 30-day wait time benchmark for cardiac rehabilitation (CR). The objectives of the current study were to: (1) describe cardiac patient perceptions of actual and ideal CR wait times, (2) describe and compare cardiac specialist and CR program perceptions of wait times, as well as whether the recommendations are appropriate and feasible, and (3) investigate actual wait times and factors that CR programs perceive to affect these wait times.

**Methods:**

Postal and online surveys to assess perceptions of CR wait times were administered to CR enrollees at intake into 1 of 8 programs, all CCS member cardiac specialists treating patients indicated for CR, and all CR programs listed in Canadian directories. Actual wait times were ascertained from the Canadian Cardiac Rehabilitation Registry. The design was cross-sectional. Responses were described and compared.

**Results:**

Responses were received from 163 CR enrollees, 71 cardiac specialists (9.3% response rate), and 92 CR programs (61.7% response rate). Patients reported that their wait time from hospital discharge to CR initiation was 65.6 ± 88.4 days (median, 42 days), while their ideal median wait time was 28 days. Most patients (91.5%) considered their wait to be acceptable, but ideal wait times varied significantly by the type of cardiac indication for CR. There were significant differences between specialist and program perceptions of the appropriate number of days to wait by most indications, with CR programs perceiving shorter waits as appropriate (p < 0.05). CR programs reported that feasible wait times were significantly longer than what was appropriate for all indications (p < 0.05). They perceived that patient travel and staff capacity were the main factors negatively affecting waits. The median wait time from referral to program initiation was 64 days (mean, 80.0 ± 62.8 days), with no difference in wait by indication.

**Conclusions:**

Wait times following access to cardiac rehabilitation are prolonged compared with consensus recommendations, and yet are generally acceptable to most patients. Wait times following percutaneous coronary intervention in particular may need to be shortened. Future research is required to provide an evidence base for wait time benchmarks.

## Background

Timely access to cardiac care is important to ensure the best patient outcomes [1]. This holds true not only for acute cardiac care [2], but also applies to outpatient cardiac care [3,4]. Indeed, there is a high risk of subsequent or recurrent events in the period after an acute cardiac hospitalization [5], and this is often a time of acute distress for patients and their families. To optimize patients’ post-discharge health and well-being, timely access to secondary prevention is required [6]. Cardiac rehabilitation (CR) is an outpatient chronic disease management program designed to improve and maintain cardiovascular health through individualized, inter-professional care. CR programs offer medical assessment, structured exercise, and client and family education, as well as comprehensive risk factor and behavior modification. It is an effective means for the secondary prevention of coronary heart disease, as evidenced by the 25% reduction in morbidity and mortality compared with usual care [7]. Based on this evidence, CR is recommended as the standard of care in clinical practice guidelines for acute coronary syndrome and revascularization [8,9], among other cardiac populations [8,10,11].

In 2004, the Canadian Cardiovascular Society (CCS) formed an Access to Care Working Group to establish reasonable, safe wait times for access to cardiovascular services and procedures [1]. Wait time benchmarks for CR services were established by cardiac indication based on clinical consensus [12]. The benchmarks specified the upper limit of both preferable and acceptable wait times, which provided a reasonable standard for physicians to treat patients based on the level of predicted risk. The overall recommendation was for a 30 calendar day “preferable” wait time from referral to start of the exercise program in CR, with 60 days as “acceptable”. In 2010, the Canadian Medical Association Wait Time Alliance published wait time benchmarks for cardiac surgery, as well as for access to cardiac services throughout the continuum, including cardiac rehabilitation (http://www.waittimealliance.ca/waittimes/cardiac_care.htm).

Little is known whether the care provided meets these recommended benchmarks, or whether key stakeholders perceive the wait times as acceptable, appropriate and feasible. In 2001, CR participants from 24 sites (45% of Ontario’s programs) were investigated, and the results showed that the mean and median times from cardiac event to CR referral were 71 and 31 days, respectively (range, 1 day to 2 years), and from the patient referral to intake to the CR program, the times were 111 and 72 days, respectively [13]. In 2003, a technology report concluded that most Canadian CR programs are running at or near capacity, and have waiting lists for admission from weeks to months [14]. Therefore, the objectives of the current study were to: (1) describe cardiac patient perceptions of actual and ideal CR wait times by cardiac indication for CR and whether they consider their wait acceptable; and (2) describe and compare cardiac specialist and CR program perceptions of the proportion of patients receiving CR within CCS-recommended wait times, as well as whether the recommendations are appropriate and feasible. Finally, with regard to CR program perceptions, the third objective was to: (a) describe program-reported CR wait times, (b) describe program awareness of wait time benchmarks, and (c) investigate factors that CR programs perceive to affect wait times.

## Methods

We present data from 3 cross-sectional studies and the Canadian Cardiac Rehabilitation Registry (www. cacr.ca/resources/registry.cfm). These studies were approved by corresponding institutional research ethics boards, as well as York University’s Office of Research Ethics. For the first study, 163 consenting CR enrollees from 1 of 8 urban and regional CR programs in Ontario participated. New patients were approached consecutively by study staff and were provided a self-report survey at program intake. Wait time items were incorporated into a survey of a larger study on inter-provider communication (detailed methods shown elsewhere; [15]).

For the second study, a survey was mailed to all 44 Ontario CR programs in early 2010. Each CR program received a personalized cover letter, questionnaire, and pre-paid return envelope. A similar anonymous online survey was sent to all 105 CR programs outside of Ontario. The instructions specified that the survey was to be completed by the most senior clinical staff member. CR programs were identified and contact information secured in collaboration with the Cardiac Rehabilitation Network of Ontario and Canadian Association of Cardiac Rehabilitation.

For the third study, an anonymous online survey was sent to all 765 cardiac specialist members of the CCS treating adult patients indicated for CR (i.e., cardiologists, cardiovascular surgeons and internists). The survey was developed in conjunction with the CCS, and was sent out by this organization to its membership in the spring of 2010. The survey was translated into French, and participants completed the survey in the language of their choice. A personalized approach with repeated contacts was used to optimize response rate for all 3 surveys.

The Canadian Cardiac Rehabilitation Registry is a national data collection system to assess the characteristics, treatments and outcomes of patients participating in CR. It is a retrospective source of consecutive participants from participating centers, with the first patients in the registry referred to CR in October 2006. Currently, there are 10 programs contributing data to the Canadian Cardiac Rehabilitation Registry, with a total of 2305 cases (n = 1568 [68.1%] males; mean age: 61.6 ± 10.6). There are an estimated 120 CR programs across the country, suggesting that the registry represents slightly < 10% of the overall CR population. Participating programs document all CR outpatients using standardized data definitions. The registry has undergone ethical and privacy review.

### Measures

The questionnaires were developed by the authors based on available literature [12,14]. Clinical input from physicians and other health care professionals with expertise in CR were incorporated during survey development. Pilot testing was undertaken with several members of the target clinical audience, which informed minor re-wording of some survey questions. All wait time items were investigator-generated, and consisted of forced-choice response options, Likert-type scales and some items called for open-ended responses.

Each survey included sociodemographic and health service/clinical items (as applicable; e.g., program characteristics, province) assessed through forced-choice response options. In the patient survey, respondents were asked approximately how many weeks passed between being discharged from hospital and starting the CR program, and between being referred to CR and starting the program. They were also asked whether they considered the wait time to be “acceptable” or “unacceptable”, and to respond why in an open-ended fashion. They were asked to check (✓) as appropriate from a list of potential reasons for delay in starting CR where applicable, if none were applicable, or if another reason was applicable (and to specify accordingly). Finally, participants were asked to report ideally how many weeks they would like to wait between being hospitalized for a cardiac event or procedure and starting CR.

In the specialist survey, respondents were asked what percentage of their eligible and their referred patients start CR within 30 days from referral. Specialists were provided a table with the CR indications, and the preferable and acceptable wait times according to Canadian consensus guidelines [12]. They were then asked to estimate the percentage of their patients meeting the preferable and acceptable benchmark. CR programs were asked to report the average wait time between patient referral and intake, and between intake and start of the exercise program in days. Similar to the cardiac specialist survey, programs were provided a table with the CR indications, and the preferable and acceptable wait times. They were then asked to estimate the percentage of patients meeting the preferable and acceptable benchmark at their respective sites, as well as their perceptions of the appropriate number of days waiting for optimal patient outcomes, and their perception of the feasible number of days waiting based on their program capacity. Finally, programs were provided with a list of patient, physician, program and health system-level factors that may contribute to wait times. Respondents were asked to what degree they perceived each factor contributed to wait times on a scale from 1 (“does not affect CR wait time delays”) to 5 (“major factor affecting wait time delays”). Finally, the Canadian Cardiac Rehabilitation Registry collects data from participating CR programs regarding the date of referral, date of referral receipt, date of initial visit and date of program start. Wait times in days were computed between these intervals.

### Statistical analyses

All data analyses were performed using IBM SPSS version 17.0 (New York, USA) [16]. A descriptive examination of the characteristics of the patients, specialists and CR programs was performed. To test the first objective, a descriptive examination of perceived and ideal patient wait times was performed. Bivariate analyses to compare wait time perceptions by patient indication were performed using t-tests. To describe cardiac specialist and CR program perceptions of the proportion of patients receiving CR within CCS-recommended wait times [12], as well as whether the recommendations are appropriate and feasible, a descriptive examination was performed. Non-parametric tests were then used to compare specialist and CR program perceptions of benchmarks and wait times. Descriptive statistics were used to describe program-reported CR wait times, program awareness of wait time benchmarks, and factors perceived to affect wait times.

## Results

### Characteristics of respondents

Self-reported characteristics of cardiac patients, cardiac specialists, and CR programs are shown in Table 1, Tables 2 and 3, respectively. Overall, 71 of 765 cardiac specialists completed the online survey (9.3% response rate). With regard to the CR programs, responses were received for 42 of 44 Ontario programs (95.4% response rate), and for 50 of 105 CR programs outside of Ontario (47.6% response rate).

**Table 1:** Characteristics of CR enrollees, and relation of clinical characteristics to ideal wait time (N = 163)

**Characteristic**	**n (%)**	**Perceived wait time from hospital discharge to CR, in days (Mean ± SD)**	**Ideal wait time, in days (Mean ± SD)**	**Association between indication and ideal wait time**^†^, t
**Sociodemographic Characteristics**				
Males (%)	128 (78.5)	-	-	-
Age (mean yrs ± SD)	62.8 ± 11.3	-	-	-
Caucasian (%)	122 (80.3)	-	-	-
Retired (%)	66 (43.4)	-	-	-
Income (% ≥ $50,000 CDN)	53 (42.1)	-	-	-
Indication / Previous Cardiovascular History*				
Percutaneous coronary intervention (%)	50 (33.3)	58.8 ± 55.8	26.4 ± 17.8	−2.66§§
Myocardial infarction (%)	49 (32.7)	60.7 ± 46.6	36.7 ± 27.7	1.1
Angina (%)	34 (22.7)	79.1 ± 116.4	27.5 ± 20.8	−1.83
Coronary artery bypass graft (%)	37 (24.7)	61.8 ± 31.9	46.9 ± 23.4	4.05§§§
Arrhythmia /pacemaker (%)	16 (10.7)	85.9 ± 67.4	50.9 ± 21.7	2.57§
Stroke (%)	14 (9.3)	70.6 ± 57.4	22.3 ± 20.5	−1.71
Heart failure (%)	8 (5.3)	210.6 ± 240.7	38.0 ± 28.0	0.49

*Note: participants were asked to self-report all previous events and procedures from those listed.

§p<0.05;§§p<0.01;§§§p<0.001.

†Compared ideal wait time to presence or absence of each listed indication using t-tests.

CR = cardiac rehabilitation.

**Table 2:** Self-reported characteristics of physician respondents (N = 71)

**Characteristic**	**n (%)**
**Specialty**	
Cardiologist	57 (80.3%)
Cardiovascular surgeon	8 (11.3%)
Internist	6 (8.5%)
**Province**	
Ontario	26 (36.6%)
Alberta	17 (23.9%)
Quebec	9 (12.7%)
British Columbia	8 (11.3%)
Saskatchewan	4 (5.6%)
New Brunswick	2 (2.8%)
Newfoundland	2 (2.8%)
Manitoba	1 (1.4%)
Nova Scotia	1 (1.4%)
Prince Edward Island	1 (1.4%)
**Urban/Rural**	
Urban	69 (97.2%)
Rural	2 (2.8%)
**Language of Survey Completed**	
English	62 (87.3%)
French	9 (12.7%)

**Table 3:** Characteristics of CR programs (N = 92)

**Element**	
Education, n (%)	89 (96.7%)
Core element of exercise, n (%)	88 (95.7%)
Risk factor identification, n (%)	85 (92.4%)
Exercise testing, n (%)	85 (92.4%)
Interprofessional team, n (%)	78 (84.8%)
Medical assessment, n (%)	70 (76.1%)
Patient Capacity*, Mean ± SD (Median)	624.6 ± 1607.0 (275)
Receive Government Funding, n (%)	56 (70.0%)

* Patient capacity refers to the number of patients the program can serve each year, in terms of staff and space.

### Cardiac patient perceptions of CR waits

The mean patient-reported wait time was 65.6 ± 88.4 days (median, 42 days) from being discharged from hospital to starting CR, and 40.1 ± 73.9 days (median, 28 days) from referral to starting CR. Patients reported that they waited from 7 days to 9.3 months from hospitalization to commencement of CR, with 54 (33.1%) commencing CR within the 30-day and 115 (64.6%) commencing within the 60-day CCS benchmark. One hundred nineteen (91.5%) patients considered their wait time acceptable. Patients reported that their ideal wait time between cardiac event/procedure and commencement of CR was 33.1 ± 22.3 days (median, 28 days). Ninety-two (56.7%) patients reported waiting longer from discharge to CR start than their ideal wait time. As shown by Student’s t-tests, patients who reported having a percutaneous coronary intervention desired significantly shorter CR waits than patients who did not have this procedure, while patients with a history of bypass surgery or arrhythmia/pacemaker desired longer waits than patients who did not have this history (Table 1).

Patients were asked to give reasons for delays in accessing CR. Twenty-one (16.9%) patients reported that they had to wait until they were well enough to participate in the program, 16 (12.9%) reported that they were waiting for their doctor to send the paperwork, 15 (12.1%) reported that there was a waiting list at the rehabilitation program, 6 (3.7%) reported that they had a trip or personal time conflict, which delayed their start in the program, 51 (41.1%) reported none of the above delays, and 12 (9.7%) reported other reasons, such as awaiting further diagnostic tests or cardiac procedures.

### Cardiac specialists’ perceptions of CR wait times

When specialists were asked whether they were aware of the CCS Access to Care CR wait time benchmarks, 29 (40.8%) responded affirmatively. Cardiac specialists perceived that 32.9% of their indicated patients started CR within 30 days from referral, and that 41.5% of their referred patients started CR within 30 days from referral. Table 4 displays specialist perceptions of the percentage of patients meeting the preferable and acceptable wait times, and perceptions of what are appropriate wait times by type of CR indication.

**Table 4:** Cardiac specialist and CR program perceptions of CR wait times in calendar days

**Indication**	**CCS wait time benchmarks**		**Specialist responses, N = 71 mean ± SD (median)**				**CR program responses, N = 92 mean ± SD (median)**		
	**Preferable**	**Acceptable**	**% of patients meeting preferable benchmark**	**% of patients meeting acceptable benchmark**	**Perception of appropriate # days wait**	**% of patients meeting preferable benchmark**	**% of patients meeting acceptable benchmark**	**Perception of appropriate # days wait**	**Perception of feasible # days wait**
CABG	21-30	30-60	25.4 ± 25.0 (20)	49.5 ± 31.5 (50)	40.0 ± 20.7 (30)	34.1 ± 36.5 (20)	63.3 ± 32.8 (73)**	30.0 ± 17.2 (30)*	43.4 ± 30.9 (42)†††
Valvular disease	21-30	30-60	25.6 ± 25.6 (20)	46.0 ± 31.6 (50)	40.2 ± 20.2 (30)	34.2 ± 36.5 (20)	64.2 ± 33.6 (75)**	29.2 ± 15.9 (30)**	42.0 ± 31.6 (41)†††
Percutaneous coronary intervention	2-7	7-60	17.7 ± 24.7 (10)	54.4 ±33.4 (60)	20.5 ± 12.6 (14)	21.8 ± 31.3 (7)	68.1 ± 32.6 (75)*	14.7 ± 12.5 (10)*	31.7 ± 35.7 (20)†††
Myocardial infarction	7-30	30-60	34.1 ± 27.7 (30)	54.0 ± 31.8 (50)	28.2 ± 18.0 (30)	37.5 ± 35.3 (30)	58.5 ± 33.1 (60)	20.6 ± 16.1 (17)*	36.0 ± 35.3 (21)†††
Heart failure	7-30	30-60	25.3 ±24.4 (20)	42.0 ± 33.2 (30)	29.0 ± 17.9 (30)	34.8 ± 35.8 (21)	58.9 ± 36.0 (60)*	17.5 ± 13.3 (14)***	30.5 ± 28.5 (22)††
Stable angina	7-30	30-60	30.1 ± 27.9 (20)	43.5 ± 32.9 (40)	34.2 ± 20.7 (30)	39.9 ± 35.2 (30)	58.1 ± 34.9 (60)*	18.9 ± 17.9 (14)***	33.6 ± 36.9 (21)††
Heart transplantation	4-10	10-60	36.1 ± 40.6 (25)	41.1 ± 40.4 (25)	43.2 ± 125.5 (21)	18.5 ± 28.6 (0)	52.9 ± 39.9 (56)	26.2 ± 19.0 (21)	34.3 ± 22.0 (30)††
Arrhythmias	1-30	30-60	41.9 ± 121.7 (20)	53.8 ± 118.5 (21)	47.4 ± 104.3 (30)	34.8 ± 35.0 (22)	57.5 ± 35.0 (60)**	18.0 ± 15.5 (14)***	36.0 ± 37.6 (21)†††

*Note: CABG = coronary artery bypass grafting; CCS = Canadian Cardiovascular Society; CR = cardiac rehabilitation.

*p < 0.05; **p < 0.01; ***p < 0.001, independent samples t-tests comparing specialists with CR program perceptions of the percentage of patients meeting preferable and acceptable benchmarks, and of the appropriate number of days’ wait.

†p < 0.05; ††p < 0.01; †††p < 0.001 for related-samples Wilcoxon signed rank tests comparing CR program perceptions of appropriate versus feasible number of days’ wait by type of cardiac indication for CR.

### Cardiac Rehabilitation program perspective

Nationally, the median wait time reported in the registry from referral to CR program start was 64 days (mean ± SD, 80.0 ± 62.8 days). There was no significant difference in wait time by indication (bypass or valve surgery, 96.5 ± 51.8 days; percutaneous coronary intervention, 93.2 ± 69.2 days; myocardial infarction/heart failure/stable and unstable angina, 97.0 ± 66.7 days; there were insufficient arrhythmia and transplant data to report reliably). Median wait times by program varied from a minimum of 36 to a maximum of 127 days. The median wait time from referral receipt to intake was 22 days (mean, 30.82 ± 38.51 days), and from intake to initiation of the exercise program was 28 days (mean, 40.98 ± 51.59 days).

CR programs perceived that a significantly greater percentage of bypass surgery, valve disease, percutaneous coronary intervention, stable angina, and heart failure patients met the acceptable wait time benchmarks compared with cardiac specialists’ perceptions (Table 4). In addition, for all indications, except for heart transplantation, CR programs perceived significantly shorter appropriate wait times than cardiac specialists. Finally, CR program perceptions of the feasible number of days’ wait was significantly greater than what was appropriate for all indications.

When CR programs were asked whether they were aware of the CCS Access to Care CR wait time benchmarks, 49 (53.8%) responded affirmatively. Forty-seven (51.1%) programs reported that they had wait time benchmarks. When programs were asked to describe them, 20 (43.5%) reported that their program has a set wait limit, 11 (23.9%) reported that their benchmarks are in accordance with the Canadian Association of Cardiac Rehabilitation (CACR) guidelines [17], 8 (17.4%) reported their program’s benchmark range, 3 (6.5%) reported that their benchmarks vary by indication, 2 (4.3%) reported that there is no wait time in their program, and 2 (4.3%) reported that they have wait time goals to the first patient phone contact only (n = 1 did not specify). Overall, programs with wait time benchmarks estimated that 75.5 ± 26.0% of the time they meet them (median, 80%).

In the CR survey, program respondents rated the degree to which factors from the patient to health system-level affect CR wait times (Table 5). Factors listed by CR programs were patient illness, preference for specific exercise times, individual readiness to participate, slow General Practitioner referrals and limited availability of stress testing.

**Table 5:** National CR program perceptions of factors affecting CR wait times (n = 92)

**Factor**	**Mean**	**Standard deviation**
Patient travel constraints, scheduling unavailability	3.7	1.2
Capacity issues: staffing	3.6	1.2
Physician initiates patient referral at outpatient visit after hospital discharge	3.6	1.2
Lack of funding	3.6	1.4
Scheduling limitations	3.5	1.3
Lack of consistent implementation of discharge order sets including CR referral	3.5	1.4
Capacity issues: operating hours	3.2	1.4
Lack of availability of alternative CR program models	3.2	1.2
Capacity issues: facility space	3.2	1.5
Geographic siting of programs	3.1	1.4
Waiting for referral information	3.0	1.3
Waiting for exercise test results	3.0	1.5
Capacity issues: lack of equipment	2.6	1.3
Patient confidentiality legislation	1.8	1.0

Note: scores range from 1 (“does not affect CR wait time delays”) to 5 (“major factor affecting wait time delays”).

CR = cardiac rehabilitation.

## Discussion

The present study is the first published national evaluation of the timeliness of access to CR, based on perceptions of patients, providers, and CR programs compared with national guidelines. Our study provides insight regarding the views and perceptions of different stakeholders on a topic that is, both in daily practice and in national policies, often determined by non-empirical assessment among a small number of individuals. There is no objective standard for acceptable wait times, other than participation rates [3,4,18,19] and some emerging evidence of a relationship between wait time and peak oxygen consumption achieved [20,21] at this time, but our analysis of the subjective views of these stakeholders has provided important information regarding the appropriateness of the consensus recommendations.

### Patient perceptions of CR waits

The current study found that, overall, the median wait for patients to access CR was 42 days, which exceeds the 30-day CCS benchmark [12] by 40%. Registry data suggests that almost half of this time was spent waiting for the referral documentation to be generated and transmitted by a healthcare provider, and just over half was spent waiting for their intake appointment to be scheduled and assessments completed to commence the individually-tailored exercise program. This finding suggests that, to reduce wait times, strategies need to be targeted to both the pre-referral process, as well as the post-referral process.

However, over 90% of patients considered their wait acceptable. Their ideal wait was 28 days, which is highly congruent with the consensus-derived CCS benchmark [12]. The most common reasons reported for delays in access were the patient’s health status, followed by physician delays in transmitting clinical information to CR. In contrast, CR programs perceived that the most common reasons for delays in access were patient unavailability for personal reasons, capacity and funding limitations, as well as delayed physician referral.

### Healthcare providers and CR waits

Overall, cardiac specialists in Canada perceived that only approximately 1/3 of their indicated patients were accessing CR within the 30-day recommended wait time overall. This varied by CR indication, with physicians perceiving that a median of 10-30% of patients meet the “preferable” benchmarks, and 21-60% of patients meet the “acceptable” benchmarks. In particular, they perceived that only 10% of percutaneous coronary intervention patients meet the preferable wait times, and 1/5-1/3 of arrhythmia, transplant and heart failure patients meet the acceptable wait times.

CR programs and specialists generally perceived that the same degree of patients were meeting the preferable CR wait times, but CR programs tended to perceive that more patients were meeting the acceptable benchmarks than the specialists. Specialist perceptions of the appropriate number of days’ of wait by CR indication were all at the high end or exceeded the CCS preferable wait time benchmarks, and were at the low end of the acceptable benchmarks. With the exception of percutaneous coronary intervention patients, it was notable that the specialists’ views on appropriate waiting times largely matched the CR programs’ views of feasible waiting times. This suggests that what is considered appropriate according to specialists could be feasible within current funding policies. CR program perceptions of the feasible number of days’ wait were significantly higher than what was considered appropriate. These findings allude to the limited capacity in the health system to deliver the highest quality care.

Overall, a patient’s median wait time from referral receipt to the start of CR in Canada is 64 days. Considering that this does not reflect the time waiting from the referral event, this exceeds the 30-day recommendation. However, the overall consensus based on patient, specialist and CR program perceptions, as well as registry data, suggests that patients generally access CR around the acceptable wait time range of 60 days in Canada.

### Strategies to reduce CR wait times

Our overall findings suggest that some patients perceive that they are experiencing prolonged CR waits, and in particular, wait times for percutaneous coronary intervention patients need to be addressed. There are 2 specific approaches that are successful in mitigating CR wait times. First, interprofessional education classes can be offered to patients shortly after hospital discharge. Provision of these sessions may encourage earlier adoption of heart health-promoting behavior, provide reassurance to patients and family members, enable verification of discharge instructions, ensure identification of any clinical issues, which may have arisen, such as infection, and mitigate some causes of wait time delays [22]. Second, other programs have liaised with inpatient cardiac wards within their institutions to facilitate initiation of CR referral before patient discharge, rather than at a post-discharge visit weeks or months later. Such referral strategies are shown to cut CR wait times in half [23]. Indeed, a recent CCS-CACR policy position recommends that all indicated patients be referred to CR systematically prior to discharge via a standard order [24].

In response to perceived access delays for patients, some CR programs have instituted innovative practices. Indeed, quality management strategies have been successfully implemented to significantly reduce wait times to access chronic disease management programming [25], such as process mapping, and performance data collection and evaluation [26]. For example, in response to delays in booking intake exercise stress tests required to initiate an exercise program, some CR programs no longer require a stress test prior to CR initiation in low-risk patients or use a six-minute walk test. Clearly, all quality management strategies need to take into consideration, not only the patient experience, but also safety and patient outcome.

### Limitations

Caution is warranted when interpreting results from the current study, mostly because of generalizability and measurement issues. There was a considerably low response to the cardiac specialist survey. Therefore, it cannot be determined whether our results are representative of cardiac specialists across the country. Respondents were primarily Ontario cardiologists in urban settings. In a review of physician response to surveys, demographic characteristics of late respondents (considered to be a proxy for non-respondents) were similar to the characteristics of respondents to the first mailing [27]. Moreover, physicians as a group are more homogeneous with regard to knowledge, training, attitudes, and behaviour than the general population, suggesting that nonresponse bias may not be as crucial in physician surveys as in surveys of the general population [27]. In addition, the response rate of patients is unknown, although CR participant response rates are usually high.

Second, respondents were mainly providing perceptions of wait times, and therefore, there might have been some error associated with the wait time estimates. Wait times were also reported from a registry in an attempt to compensate for this limitation. However, it remains unknown whether different stakeholders may be over or under-estimating waits. Third, the multiple comparisons made between cardiac specialist and CR program perceptions may have resulted in inflated error rates. A more conservative approach was not adopted given the lack of information known in this domain; therefore, further study is warranted. Finally, the cross-sectional design precludes causal conclusions.

## Conclusions

In conclusion, wait times for access to CR may be prolonged compared with consensus recommendations [12], but they are generally acceptable to most patients. The differing views of stakeholder groups regarding acceptability and appropriateness of wait times and the cause of delays highlight the need for further research. Clearly, the timing is right to establish an evidence base for wait time benchmarks in Canada, as well as for other countries.

## Competing interests

The authors declare that they have no competing interests.

## Authors’ contributions

SG made substantial contributions to conception, design, acquisition and interpretation of data, and drafting the manuscript. YT undertook data acquisition and analysis, as well as drafting the manuscript. LM made substantial contributions to conception and design, as well as interpretation of data. WD made substantial contributions to conception and design, as well as interpretation of data. CS was involved in acquisition and interpretation of data. NS made substantial contributions to conception and design. CC critically revised the manuscript for important intellectual content and gave final approval of the version to be published. All authors read and approved the final manuscript.

## Pre-publication history

The pre-publication history for this paper can be accessed here:

http://www.biomedcentral.com/1472-6963/12/259/prepub
